# Association between Time of Day of Sports-Related Physical Activity and the Onset of Acute Myocardial Infarction in a Chinese Population

**DOI:** 10.1371/journal.pone.0146472

**Published:** 2016-01-11

**Authors:** Shan Zhao, Zhen Zhang, Qingqing Long, Yao Ma, Xiaoqing Lian, Yang Yang, Wei Gao, Zhong Chen, Liansheng Wang

**Affiliations:** 1 Department of Cardiology, the First Affiliated Hospital of Nanjing Medical University, Nanjing, 210029, China; 2 Department of Cardiology, Shanghai Jiao Tong University Affiliated Sixth People’s Hospital, No. 600 Yishan Road, Shanghai, 200233,China; Center for Interdisciplinary Research in Biology (CIRB), FRANCE

## Abstract

**Objective:**

To investigate the association between the time of day of sports-related physical activity and the onset of acute myocardial infarction (AMI) in a coronary artery disease (CAD) population in China.

**Methods:**

Between February 2014 and March 2015, a total of 696 patients from Nanjing, China, who had CAD were studied and divided into two groups (Non-AMI and AMI groups). The work-related activity and sports-related physical activity information were obtained from a self-reporting predesigned patient questionnaire.

**Results:**

Sports-related physical activity was associated with a lower risk of the onset of AMI, after adjusting the established and potential confounders, with an adjusted odds ratio (OR) of 0.67 (95% CI, 0.47–0.94) compared with those who did not have any sports-related physical activity. A dose–response relationship was observed for intensity, duration, and frequency of sports-related physical activity. Further stratification analysis revealed that the protective effects of sports-related physical activity were significant in the morning and evening groups, and patients who exercised in the evening were at a lower risk of AMI than those doing sports-related physical activity in the morning. The adjusted ORs for doing sports-related physical activity in the morning and evening groups were 0.60(0.36–0.98) and 0.56(0.37–0.87), respectively, compared with inactivity (all *P*<0.05). On the occurrence of AMI, doing sports-related physical activity in the evening had an adjusted OR of 0.93 (95% CI, 0.54–1.64, *P* = 0.824) compared with in the morning group.

**Conclusions:**

Sports-related physical activity is associated with a lower risk of onset of AMI than inactivity in Chinese people. For CAD patients, we suggest they participate in sports-related physical activity of high intensity, long duration, and high frequency. Doing sports-related physical activity in the evening and in the morning have similar benefits on the prevention of the onset of AMI.

## Introduction

Worldwide, coronary artery disease (CAD) is the single most frequent cause of death. Nearly 7.4 million people die from CAD every year, accounting for 13.1% of all deaths [1]. Furthermore, in patients with CAD, acute myocardial infarction (AMI) is the leading cause of cardiac death.

Substantial amounts of research indicate that physical activity has been associated with a reduced risk of CAD [2–5]. Physical activity not only decreased mortality in patients with established CAD, but also increased survival of CAD patients who participated in rehabilitation programs [6,7]. Although the benefit of physical activity is recognized, studies still have shown that physical activity is related to sudden cardiac death (SCD), even in a healthy population [8], and this problem worries people, especially patients with CAD.

In the past few decades, evidence has been found to support the clinical significance of circadian variation with the onset of MI and SCD [9–12]. The study by Kanaley et al [13] reported that exercising in the morning has a greater potential for inducing SCD and myocardial ischemia, and it may be sensible for patients with CAD not to exercise at this time. Our previous study indicates that the protective effect of exercise in the evening is greater than that in the morning [14]. Patients with CAD who exercised in the evening have achieved greater improvement in lipids and inflammatory markers than those exercising in the morning [15]. However, the effects of sports-related physical activity on the onset of AMI is not clear.

The present study aimed to explore the association between sports-related physical activity and the onset of AMI in a CAD population, including the intensity, duration, frequency of exercise, and the time of day of sports-related physical activity.

## Materials and Method

The study protocol was approved by the Ethics Committee of the First Affiliated Hospital of Nanjing Medical University (Nanjing, China). Written informed consent was obtained from each patient.

### Subjects

We selected all patients younger than 80 years of age who underwent coronary angiography and had at least one coronary artery with >50% luminal diameter stenosis on angiography at two hospitals (the First Affiliated Hospital of Nanjing Medical University and Nanjing Drum Tower Hospital) from February 2014 to March 2015 in Nanjing, China. All patients underwent coronary arteriography because of typical or atypical chest pain, or abnormal ST–T changes. The First Affiliated Hospital of Nanjing Medical University and Nanjing Drum Tower Hospital are two of the largest comprehensive hospitals in Nanjing and also are hospitals designated for coronary angiography according to medical insurance guidelines. Most patients who need coronary angiography in Nanjing go to these two hospitals for treatment. Patients were excluded if they had previously undergone coronary angiography or postrevascularization of the coronary arteries or had valvular heart disease, cardiomyopathy, or myocarditis. Patients with stroke sequelae, pulmonary infection, renal failure, cancer and lower limb disabilities who were not suitable for physical activity were also excluded to avoid subjective bias.

### Study design

A case-control design was used to control the confounding effect of some demographic factors (age, area of residence) that are risk factors for CAD. A total of 696 patients with CAD were divided into two groups: Non-AMI group (n = 348) and AMI group (n = 348). The patients without AMI and prior MI were defined as controls.

MI was defined by detection of a rise and/or fall in cardiac biomarker values (preferably troponin) with at least one value above the 99th percentile of the upper reference limit and with at least one of the following: symptoms of ischemia; new or presumably new significant ST-T changes or new LBBB; development of pathological Q waves in the ECG; imaging evidence of new loss of viable myocardium, or new regional wall motion abnormality; identification of an intracoronary thrombus by angiography or autopsy.

Participants were defined as exercisers if they have done sports-related physical activity, which is a subcategory of physical activity, that is planned, structured, repetitive, and aims to improve or maintain one or more components of physical fitness, for at least 5 years, and still exercised in the recent 3 months before they went to hospital.

### Measurements

#### Measures of physical activity

Sports-related physical activity was assessed according to the Baecke physical activity questionnaire (BPAQ) [16]. Answering the questionnaire, the patients self-reported, the intensity, duration, and frequency of sports-related physical activity. All participants were asked, “what kind of sport do you play most frequently?” We provided 14 types of common Chinese sports to choose from, including walking, bicycling (slow or fast), jogging, square dancing, swimming (slow or fast), aerobic exercise, table tennis, badminton, tennis, basketball, tai chi, and flexibility exercises. Each type of physical activity has activity codes and metabolic equivalent (METs) intensity [17], which is used to quantify the types of physical activity that characterize high-intensity (>6METs), moderate-intensity (3 to 6METs), and low-intensity (<3METs) [18]. The duration of each time (including warm-up time), and the frequency of exercise each week were self-reported on the questionnaire. Total energy expenditure in physical activity (EEPA) was used for the combination of intensity, duration and frequency of sports-related physical activity, the unit used to quantify the amount of total physical activity was MET·min/week [19].

Work-related activity was identified by asking patients about their main occupation, present or past. This type of activity was classified into three categories: “low” was physically very easy, sitting office work; “moderate” was work including standing and walking, light industrial worker; “high” was work including walking and lifting, or heavy manual labor [20].

#### Assessment of time of day

In reference to Chinese life routines, each day was divided into 6 sessions according to the 24-hour clock. Patients were grouped according to the time of their regular exercise. Patients who exercised mainly between 6:00–10:00 were defined as the morning group, 10:00–14:00 noon group, 14:00–18:00 afternoon group, 18:00–22:00 evening group, 22:00–2:00 night group, 2:00–6:00 early morning group.

#### Data collection

Data were collected by face-to-face interview with the consent of the patients. All of the interviewers were professionals and trained uniformly by the first author. All patients were interviewed before coronary arteriography was carried out, therefore, both interviewers and patients neither knew the result of the coronary angiography nor the relationship between time of day of physical activity and the onset of AMI. Habitual physical activity was assessed before a “reference date,” defined as 5 years before the coronary angiography both in cases and controls. A structured questionnaire was used to collect the required information on family history of CAD (any kind of CAD in first-degree relatives), medical history, current medications, and CAD risk factors, such as a history of hypertension, diabetes mellitus, dyslipidemia, cigarette smoking, and alcohol intake.

Total cholesterol (TC), high-density lipoprotein (HDL), Low-density lipoprotein (LDL), and fasting blood glucose levels were collected from the patients’ most recent medical records. Patients were considered to have dyslipidemia if their serum TC >200 mg/dL (5.18 mmol/L); or LDL ≥130 mg/dL (3.37 mmol/L); HDL <40 mg/dL (1.04 mmol/L) in men and <50 mg/dL (1.30 mmol/L) in women; or Currently receiving antilipidemic treatment[21]. Diabetes mellitus was defined as Hemoglobin A1c ≥6.5%; or Fasting plasma glucose ≥126 mg/dL (7.0 mmol/L); or 2-h Plasma glucose ≥200 mg/dL (11.1 mmol/L) during an oral glucose tolerance test; or In a patient with classic symptoms of hyperglycemia or hyperglycemic crisis, a random plasma glucose ≥200 mg/dL (11.1 mmol/L) or with the use of hypoglycemic treatment[21]. Resting blood pressure was measured using an automated sphygmomanometer and was recorded as the average of 3 seated measurements taken 5 min apart. A current diagnosis of hypertension was defined by any 1 of the following: History of hypertension diagnosed and treated with medication, diet, and/or exercise; Prior documentation of blood pressure ≥140 mmHg systolic and/or 90 mmHg diastolic for patients without diabetes or chronic kidney disease, or prior documentation of blood pressure ≥130 mmHg systolic or 80 mmHg diastolic on at least 2 occasions for patients with diabetes or chronic kidney disease; Currently undergoing pharmacological therapy for treatment of hypertension[21]. The degree of coronary stenosis was assessed in the worst view projection. All patients were divided into 4 groups according to the maximum extent of coronary stenosis: stenosis extent (50%-74%), stenosis extent (75%-90%), stenosis extent (91%-99%), and stenosis extent (100%).

### Statistical analysis

Data were analyzed using the Statistics Package for Social Sciences (v16.0; SPSS Inc., Chicago, IL, USA). In this study, age and BMI data were treated as continuous variables and presented as mean ± SD. Comparisons of mean values of nonnormal continuous variables by clinical outcome (age and BMI) were performed using the Mann–Whitney U-test. Categorical variables were tested by the calculation of the chi-square test. The logistic regression analysis was used to estimate the crude and adjusted odds ratio (OR) of the onset of AMI for different levels (intensity, duration, frequency) and different times of day of sports-related physical activity. We estimated the 95% confidence interval (CI) of OR. Differences were considered significant if *P*-value ≤0.05. All *P*-values were 2-tailed.

## Results

### Subjects

Table 1 presents the characteristics of 696 patients. Compared with their controls, AMI patients had lower left ventricular ejection fraction (LVEF) (%) (62.9±5.2 vs 55.4±10.8, *P*<0.01) and more severe coronary artery stenosis (*P*<0.001). The non-AMI patients were more likely to have hypertension and diabetes mellitus (*P*<0.05). In addition, subjects with AMI were more likely to have a family history of CAD (*P*<0.05) and had a higher prevalence of smoking (*P*<0.001). The work-related activity of the non-AMI group was less than that in the AMI group. No statistical significance was found in alcohol drinking, body mass index (kg/m^2^), dyslipidemia and the number of stenosis vessels between the AMI group and control group. The vessel disease status of 696 patients is shown in Table 2. No significant difference was found in coronary artery stenosis between the exerciser group and non-exerciser group. A higher proportion of single vessel disease status was distributed to the exerciser group while a higher proportion of multi vessel disease status was distributed to the non-exerciser group (*P*<0.01). Characteristics of time of day of sports-related physical activity are shown in Table 3. The proportions of intensity and duration time of sports-related physical activity in subgroups were not statistically significant. A higher proportion of “>5 times/week” was distributed to the morning group while a lower proportion of “>5times/week” was distributed to the noon group (*P* <0.01).

**Table 1:** Characteristics of Patients With and Without AMI.

Characteristic variables	Non-AMI(%)(N = 348)	AMI(%)(N = 348)	*P* Value
**Age**	64.2±9.3	62.7±11.7	0.057
**LVEF**	62.9±5.2	55.4±10.8	<0.001
**Sex**			0.021
Male	252(72.4)	278(79.9)	
Female	96(27.6)	70(20.1)	
**Body mass index(kg/m**^**2**^**)**	25.0±3.0	24.6±3.4	0.090
**Family history of CAD**	40(11.5)	62(17.8)	0.024
**Hypertension**	232(66.7)	183(52.6)	<0.001
**Diabetes mellitus**	93(26.7)	67(19.3)	0.019
**Dyslipidemia**	279(80.2)	250(71.8)	0.854
**Smoking status**			<0.001
Never	186(53.4)	128(36.8)	
Former	48(13.8)	41(11.8)	
Current<20 cigarettes	42(12.1)	54(15.5)	
Current≥20 cigarettes	72(20.7)	125(35.9)	
**Alcohol drinking**			0.563
Never	240(69.0)	247(70.9)	
Former	22(6.3)	27(7.8)	
Current	86(24.7)	74(21.3)	
**Work-related Activity**			0.029
Low	285(81.9)	275(79.1)	
Moderate	18(5.2)	28(8.0)	
High	45(12.9)	45(12.9)	
**Coronary stenosis (%)**			<0.001
50~74	70(20.1)	18(5.2)	
75~90	153(44.0)	124(35.6)	
91~99	85(24.4)	90(25.9)	
100	40(11.5)	116(33.3)	
**The number of stenosis vessels (N)**			0.114
1	121(34.8)	96(27.6)	
2	94(27.0)	109(31.3)	
3	133(38.2)	143(41.1)	

Abbreviations: AMI, acute myocardial infarction; CAD, coronary artery disease; LVEF, left ventricular ejection fraction; Continuous values expressed as mean ± SD; categorical variables expressed as N (%).

**Table 2:** Vessel Disease Status of Exerciser and Non-exerciser.

Vessel disease status	Exerciser ^a^ (N = 330)	Non-exerciser (N = 366)	*P* Value
**Coronary stenosis (%)**			0.888
50~74	44(13.3)	44(12.0)	
75~90	133(40.3)	144(39.3)	
91~99	79(23.9)	96(26.2)	
100	74(22.4)	82(22.4)	
**The number of stenosis vessels (N)**			0.003
1	121(36.7)	96(26.2)	
2	79(23.9)	124(33.9)	
3	130(39.4)	146(39.9)	

Categorical variables expressed as N (%).

^a^ Participants were defined as exercisers if they have done sports-related physical activity, which is a subcategory of physical activity, that is planned, structured, repetitive, and aims to improve or maintain one or more components of physical fitness, for at least 5 years, and still exercised in the recent 3 months before they went to hospital.

**Table 3:** Characteristics of Time of Day of Sports-related Physical Activity.

Sports-relatedphysical activity	Morning (N = 121)	Noon(N = 6)	Afternoon (N = 49)	Evening (N = 158)	Early morning (N = 32)	*P* Value
**Intensity** ^a^						0.246
Low	79(65.3)	3(50.0)	37(75.5)	110(69.6)	19(59.4)	
Moderate	20(16.5)	0(0)	7(14.3)	16(10.1)	6(18.8)	
High	22(18.2)	3(50.0)	5(10.2)	32(20.3)	7(21.9)	
**Duration time**						0.382
<30 min/day	10(8.3)	1(16.7)	5(10.2)	11(7.0)	1(3.1)	
30~60min/day	42(34.7)	3(50.0)	24(49.0)	61(38.6)	9(28.1)	
>60 min/day	69(57.0)	2(33.3)	20(40.8)	86(54.4)	22(68.8)	
**Frequency**						0.009
<3 times/week	7(5.8)	1(16.7)	4(8.2)	19(12.0)	1(3.1)	
3~5 times/week	3(2.5)	1(16.7)	9(18.4)	20(12.7)	2(6.2)	
>5 times/week	111(91.7)	4(66.7)	36(73.5)	119(75.3)	29(90.6)	

Categorical variables expressed as N (%).

^a^ Intensity of sports-related physical activity was categorized as low (<3METs), moderate (3–6METs) and high (>6METs).

### Sports-related physical activity and the onset of AMI

Table 4 shows that exercisers had a decreased risk of AMI with a crude OR of 0.64 (95%CI, 0.48–0.87, *P*<0.05) and an adjusted OR of 0.67 (95%CI, 0.47–0.94, *P*<0.05), compared with those who never participated in physical activity.

**Table 4:** Association Between Sports-related Physical Activity and the Onset of AMI.

Sports-related physical activity	Non-AMI (%)	AMI (%)	Unadjusted OR (95%CI)	Adjusted OR^c^ (95%CI)	*P* Value
**Exerciser** ^a^					
No	146(42.0)	184(52.9)	1(reference)	1(reference)	
Yes	202(58.0)	164(47.1)	0.64(0.48–0.87)	0.67(0.47–0.94)	0.023
**Intensity** ^b^					
None	146(42.0)	184(52.9)	1(reference)	1(reference)	
Low	131(37.6)	118(33.9)	0.71(0.51–0.99)	0.80(0.55–1.18)	0.259
Moderate	26 (7.5)	22(6.3)	0.67(0.37–1.23)	0.60(0.30–1.20)	0.150
High	45(12.9)	24(6.9)	0.42(0.25–0.73)	0.39(0.22–0.73)	0.003
**Duration time**					
None	146(42.0)	184(52.9)	1(reference)	1(reference)	
<30 min/day	14(4.0)	14(4.0)	0.79(0.37–1.72)	0.81(0.34–1.93)	0.637
30~60min/day	80(23.0)	59(17.0)	0.59(0.39–0.87)	0.65(0.41–1.02)	0.062
>60 min/day	108(31.0)	91(26.1)	0.67(0.47–0.95)	0.67(0.45–0.99)	0.048
**Frequency**					
None	146(42.0)	184(52.9)	1(reference)	1(reference)	
<3 times/week	11(3.2)	21(6.0)	1.52(0.71–3.25)	1.87(0.79–4.42)	0.155
3~5 times/week	21(6.0)	14(4.0)	0.53(0.26–1.08)	0.94(0.43–2.05)	0.869
>5 times/week	170(48.9)	129(37.1)	0.60(0.44–0.83)	0.57(0.39–0.82)	0.003
**Total EEPA**					
None	146(42.0)	184(52.9)	1(reference)	1(reference)	
1~450 MET·min/week	14(4.0)	22(6.3)	1.25(0.62–2.52)	1.64(0.73–3.64)	0.229
451~900 MET·min/week	68(19.5)	48(13.8)	0.56(0.36–0.86)	0.60(0.36–0.97)	0.039
901~1350 MET·min/week	62(17.8)	67(19.3)	0.86(0.57–1.29)	0.88(0.55–1.41)	0.603
1351~1800 MET·min/week	31(8.9)	18(5.2)	0.46(0.25–0.86)	0.45(0.23–0.90)	0.025
>1800 MET·min/week	27(7.8)	9(2.6)	0.26(0.12–0.58)	0.18(0.07–0.45)	<0.001

Abbreviations: AMI, acute myocardial infarction; OR, odds ratio; CI, confidence interval; EEPA, energy expenditure in physical activity.

^a^ Participants were defined as exercisers if they have done sports-related physical activity, which is a subcategory of physical activity, that is planned, structured, repetitive, and aims to improve or maintain one or more components of physical fitness, for at least 5 years, and still exercised in the recent 3 months before they went to hospital.

^b^ Intensity of sports-related physical activity was categorized as low (<3METs), moderate (3–6METs) and high (>6METs).

^c^ Adjustment for age, sex, smoking status, alcohol use, work-related activity, hypertension, dyslipidemia, diabetes mellitus, family history of CAD, the severity of coronary stenosis and body mass index in the analysis.

The crude and adjusted OR for high-intensity sports-related physical activity were 0.42 (95%CI, 0.25–0.73, *P*<0.01) and 0.39 (95%CI, 0.22–0.73, *P*<0.01) compared with those who never participated in physical activity. There was no statistical significance in low-intensity and moderate-intensity sports-related physical activity between the AMI and control groups.

The risk of AMI decreased while sports-related physical activity duration time was >60 min/day, the adjusted OR was 0.67 (95%CI, 0.45–0.99, *P*<0.05). When sports-related physical activity frequency was >5 times/week, patients had a decreased risk of AMI with a crude OR of 0.60 (95%CI, 0.44–0.83, *P*<0.01) and an adjusted OR of 0.57 (95%CI, 0.39–0.82, *P*<0.01), compared with inactivity.

Total energy expenditure in physical activity (EEPA) between 451 and 900 MET·min/week had a decreased risk of AMI with adjusted OR of 0.60 (95%CI, 0.36–0.97, *P*<0.05), compared with inactivity. Further increases in total EEPA were also associated with lower OR of AMI odds.

### Different times of day of sports-related physical activity and the onset of AMI

The relationship between time of day of sports-related physical activity and AMI are shown in Table 5. After adjusting for age, sex, smoking status, alcohol use, hypertension, dyslipidemia, diabetes mellitus, family history of CAD, the severity of coronary stenosis, body mass index, and work-related activity, the results interestingly show that exercising at different times of the day was significantly associated with the onset of AMI. The adjusted ORs of doing sports-related physical activity in the morning (6:00–10:00) and evening (18:00–22:00) groups were 0.60 (95%CI, 0.36–0.98) and 0.56 (95%CI, 0.37–0.87), respectively, compared with inactivity (*P*<0.05). The adjusted OR of doing sports-related physical activity in the evening group was 0.93 (95%CI, 0.54–1.64), compared with morning group (*P>*0.05).

**Table 5:** Association Between Different Times of Day Sports-related Physical Activity and the Onset of AMI.

Sports-related physical activity ^a^	Non-AMI (%)	AMI (%)	Unadjusted OR (95%CI)	Adjusted OR ^b^ (95%CI)	*P* Value
**None**	146(42.0)	184(52.9)	1(reference)	1(reference)	
**Morning (6:00–10:00)**	73(21.0)	48(13.8)	0.52(0.34–0.80)	0.60(0.36–0.98)	0.042
**Noon (10:00~14:00)**	0(0.0)	6(1.7)	--	--	--
**Afternoon (14:00–18:00)**	25(7.2)	24(6.9)	0.76(0.42–1.39)	0.87(0.44–1.71)	0.685
**Evening (18:00–22:00)**	89(25.6)	69(19.8)	0.61(0.42–0.90)	0.56(0.37–0.87)	0.009
**Night (22:00–2:00)**	0(0.0)	0(0.0)	--	--	--
**Early morning (2:00–6:00)**	15(4.3)	17(4.9)	0.90(0.43–1.86)	0.98(0.43–2.27)	0.973
**Morning (6:00–10:00)**	73(21.0)	48(13.8)	1(reference)	1(reference)	
**Evening (18:00–22:00)**	89(25.6)	69(19.8)	1.18(0.73–1.91)	0.93(0.54–1.64)	0.824

Abbreviations: AMI, acute myocardial infarction; OR, odds ratio; CI, confidence interval.

^*a*^ Participants were defined as exercisers if they have done sports-related physical activity, which is a subcategory of physical activity, that is planned, structured, repetitive, and aims to improve or maintain one or more components of physical fitness, for at least 5 years, and still exercised in the recent 3 months before they went to hospital.

^*b*^ Adjustment for age, sex, smoking status, alcohol use, work-related activity, hypertension, dyslipidemia, diabetes mellitus, family history of CAD, the severity of coronary stenosis, and body mass index in the analysis.

## Discussion

The present study evaluated the association between sports-related physical activity and the onset of AMI in a Chinese population. The results show that sports-related physical activity is inversely associated with the onset of AMI. AMI risk decreased with the increase in intensity, duration, and frequency of sports-related physical activity. To the best of our knowledge, this research is the first report of the association between time of day of sports-related physical activity and the incidence of AMI in China.

In the past few decades, much research has proven that regular physical activity has a beneficial effect on the reduction of CAD risk. But few studies have explored the association between physical activity and the onset of AMI. Previous studies focused on the effects of total physical activity and work-related activity, but did not give enough attention to sports-related physical activity. Sports-related physical activity is also defined as exercise, which is a subcategory of physical activity, that is planned, structured, repetitive, and aims to improve or maintain one or more components of physical fitness [22]. This study shows that sports-related physical activity is associated with a lower risk of onset of AMI than inactivity in Chinese people. Participating in sports-related physical activity of high intensity, long duration, and high frequency may protect CAD patients from the occurrence of AMI. Unexpectedly, this research showed that high-intensity exercise did not cause the occurrence of AMI, but helped to prevent the onset of AMI.

We also explored the possible triggers of the onset of AMI, and high-intensity physical activity (>6 METS) before onset of MI symptoms was reported in only 4 (1.1%) patients, which showed that high-intensity physical activity was not the main cause of the onset of AMI. The present report supports the findings of many other studies that habitual heavy physical activity [23] or vigorous exercise [24–28] is associated with significantly reduced risk of all cause, cardiovascular disease (CVD), and CAD death. Rognmo et al. also indicated that the risk of a cardiovascular event was low after both high-intensity exercise and moderate-intensity exercise in a cardiovascular rehabilitation setting [29].

Considering that exercise at higher relative intensities has been found to provoke a greater increase in aerobic capacity and greater cardioprotective effects than exercise at moderate intensities, high-intensity exercise should be considered among patients with CAD. Long-term high-intensity exercise helped to reduce the risk of AMI, but a sudden vigorous exercise might increase the risk of AMI. So gradually progress, instead of a sudden increase in the intensity of exercise was recommended for CAD patients, especially sedentary patients. In China, people prefer low-intensity exercise (such as walking slowly after meals). According to the results of the present study, high-intensity exercise had a more positive effect on prevention of the onset of AMI compared to low-moderate intensity exercise. So we suggest that Chinese people do higher intensity exercises, such as walking at a brisk pace and jogging.

The protection of AMI associated with regular exercise is rooted in its control of many risk factors, including obesity [30], blood pressure [31,32], diabetes mellitus [33], triglycerides, low-density lipoprotein cholesterol (LDL-C), high-density lipoprotein cholesterol (HDL-C) [34], and smoking cessation [35]. Furthermore, exercise has been shown to inhibit hemostatic function and inflammatory markers, oxidative stress, to improve VO_2_peak and aerobic capacity [36,37], and to favorably modulate plasma superoxide dismutase activity and endothelial function [38,39]. High-intensity exercise has been found more effective for improving VO_2_peak, aerobic capacity and endothelial function than low-intensity and moderate-intensity exercise. Long-term exercise, especially high-intensity exercise, helped to reduce the wall stress by lowering blood pressure, to decrease coronary artery spasm, and to raise the threshold of the intensity of exercise that would induce myocardial ischemia, further, to reduce risk of the onset of AMI.

In previous research, evidence has been found to support the clinical significance of circadian variation with the onset of unstable angina, MI, and SCD [9–12]. Unstable angina [10] and MI [9] onset occurrence peaks between 6 am and noon. The Framingham Heart Study showed that there was a pronounced circadian variation in the occurrence of definite or possible SCD with a peak incidence between 7 and 9 am, and MI occurred in about one-third of all cases of SCD [11]. It is unclear whether morning exercise is different from physical activity performed at other times of day. Murray et al [40] assigned patients with established CAD to a morning (07:30) or afternoon (15:00) training group participating in long-term submaximal exercise to explore this hypothesis, but no statistically significant result was found. Zhao et al [14] reported that the protective effect of exercise in the evening was greater than that in the morning. Lian et al [15] found that patients with CAD who walked in the evening had greater improvements in lipids and inflammatory markers than those walking in the morning. Until now, however, no research has found that exercise in the morning has an adverse effect on CAD or AMI.

In the present study, the adjusted ORs of exercising in the morning (6:00–10:00) and evening (18:00–22:00) groups were 0.60 and 0.56, respectively, compared with inactivity. Patients with CAD exercising in the evening seemed to have a lower risk ratio of AMI compared with those exercising in the morning. The proportions of intensity and duration time of sports-related physical activity in morning group and evening group were not statistically significant. While exercisers in morning group exercised more frequent than exercisers in evening group, which might underestimate the benefits of exercising in the evening. These findings further explored the prevention of exercise in the onset of AMI. The direct mechanism causing exercising at different times of the day to prevent the onset of AMI is unclear. Previous studies show that the response of ambulatory blood pressure to everyday physical activities was highest between 08:00 and 10:00 hours [41,42], and greater shear stress may be evident after morning exercise [43]. The tone of the large coronary artery is higher and its diameter is smaller in the early morning than in the afternoon and evening [44]. Coronary spasm also has a circadian variation and occurs predominantly from midnight to early morning and is usually not easy to be induced by exercise in the daytime [44–47]. Flow-mediated vasodilation in patients with variant angina deteriorated by the early morning (6 am) and improved by the afternoon (2 pm) and evening (8 pm) [46]. Morikawa proved that 3-day long aerobic interval exercise in the afternoon suppressed the attacks and improved the endothelial function, oxidative stress, inflammation, and insulin resistance in patients with coronary spastic angina [48]. Lian et al. reported that patients with CAD walking in the evening have greater improvements in lipids and inflammatory markers than those walking in the morning [15]. Doing sports-related physical activity in the evening may provide greater improvement in fibrinogen, LDL-C, white blood cell (WBC) count, and high sensitivity C-reactive protein (hsCRP) compared with this activity in the morning. And doing sports-related physical activity in the evening may avoid the potentially adverse physiologic changes, such as morning blood pressure surge (MBPS), increases in platelet aggregation, fibrinogen, catecholamine and cortisol levels in the morning, and an antifibrinolytic tendency in the early morning, which would increase plaque vulnerability, disruption and thrombosis, and lead to the onset of AMI. The present research found no exercise in the morning has induced AMI, so we suggest that CAD patients do not have to avoid sports-related physical activity in the morning. It is more likely that participants will gain benefits from sports-related physical activity, no matter what time of day.

### Study strengths and limitations

Several potential limitations of our study must be considered when interpreting the results. First, the relatively small sample size of this study may limit the statistical power. Second, the study was limited by its retrospective design, and potential recall bias. Although the selection was conducted carefully, the inherent selection bias cannot be eliminated completely. This kind of bias should be avoided in further studies. Third, we did not obtain detailed information on medication use predisposing to the risk of the onset of AMI, therefore, we cannot eliminate the possibility that medication factors confounded the association between sports-related physical activity and the onset of AMI. Finally, the patients included in our study were Chinese only; therefore, the results should be extrapolated to other populations with caution.

## Conclusions

Our findings first demonstrate that sports-related physical activity is inversely associated with the onset of AMI in a Chinese population. Further more, doing sports-related physical activity in the evening and in the morning have similar benefits on the prevention of AMI.

## Supporting Information

S1 Table Characteristics of Patients With and Without AMI. (DOCX)

S2 Table Vessel Disease Status of Exerciser and Non-exerciser. (DOCX)

S3 Table Characteristics of Time of Day of Sports-related physical activity. (DOCX)

S4 Table Association Between Sports-related Physical Activity and the Onset of AMI. (DOCX)

S5 Table Association Between Different Times of Day Sports-related Physical Activity and the Onset of AMI. (DOCX)

S6 Table PLOSOne_Clinical_Studies_Checklist. (DOCX)

## Abbreviations

AMI: acute myocardial infarction
BMI: body mass index
BPAQ: Baecke physical activity questionnaire
CAD: coronary artery disease
CVD: cardiovascular disease
ECG: electrocardiograph
EEPA: energy expenditure in physical activity
HDL-C: high-density lipoprotein cholesterol
hsCRP: high sensitivity C-reactive protein
LBBB: left bundle branch block
LDL-C: low-density lipoprotein cholesterol
LVEF: left ventricular ejection fraction
MBPS: morning blood pressure surge
METs: metabolic equivalent
OR: odds ratio
SCD: sudden cardiac death
TC: total cholesterol
TG: triglyceride
WBC: white blood cell
