# Supplementary material for: Association between Time of Day of Sports-Related Physical Activity and the Onset of Acute Myocardial Infarction in a Chinese Population
Source: PLoS One. 2016 Jan 11;11(1):e0146472. doi: 10.1371/journal.pone.0146472 (PMC4709000; doi:10.1371/journal.pone.0146472)
Supplement: S1 Table — (DOCX) [file pone.0146472.s001.docx]

Table 1. Characteristics of Patients With and Without AMI

| **Characteristic variables** | **Non-AMI(%)(N=348)** | **AMI(%)(N=348)** | ***P* Value** |
| --- | --- | --- | --- |
| **Age** | 64.2±9.3 | 62.7±11.7 | 0.057 |
| **LVEF** | 62.9±5.2 | 55.4±10.8 | <0.001 |
| **Sex** |  |  | 0.021 |
| Male | 252(72.4) | 278(79.9) |  |
| Female | 96(27.6) | 70(20.1) |  |
| **Body mass index(kg/m^2^)** | 25.0±3.0 | 24.6±3.4 | 0.090 |
| **Family history of CAD** | 40(11.5) | 62(17.8) | 0.024 |
| **Hypertension** | 232(66.7) | 183(52.6) | <0.001 |
| **Diabetes mellitus** | 93(26.7) | 67(19.3) | 0.019 |
| **Dyslipidemia** | 279(80.2) | 250(71.8) | 0.854 |
| **Smoking status** |  |  | <0.001 |
| Never | 186(53.4) | 128(36.8) |  |
| Former | 48(13.8) | 41(11.8) |  |
| Current<20 cigarettes | 42(12.1) | 54(15.5) |  |
| Current≥20 cigarettes | 72(20.7) | 125(35.9) |  |
| **Alcohol drinking** |  |  | 0.563 |
| Never | 240(69.0) | 247(70.9) |  |
| Former | 22(6.3) | 27(7.8) |  |
| Current | 86(24.7) | 74(21.3) |  |
| **Work-related Activity** |  |  | 0.029 |
| Low | 285(81.9) | 275(79.1) |  |
| Moderate | 18(5.2) | 28(8.0) |  |
| High | 45(12.9) | 45(12.9) |  |
| **Coronary stenosis (%)** |  |  | <0.001 |
| 50~74 | 70(20.1) | 18(5.2) |  |
| 75~90 | 153(44.0) | 124(35.6) |  |
| 91~99 | 85(24.4) | 90(25.9) |  |
| 100 | 40(11.5) | 116(33.3) |  |
| **The number of stenosis vessels (N)** |  |  | 0.114 |
| 1 | 121(34.8) | 96(27.6) |  |
| 2 | 94(27.0) | 109(31.3) |  |
| 3 | 133(38.2) | 143(41.1) |  |

Abbreviations: AMI, acute myocardial infarction; CAD, coronary artery disease; LVEF, left ventricular ejection fraction.

Continuous values expressed as mean + SD; categorical variables expressed as N (%).
