# Supplementary material for: Association between Time of Day of Sports-Related Physical Activity and the Onset of Acute Myocardial Infarction in a Chinese Population
Source: PLoS One. 2016 Jan 11;11(1):e0146472. doi: 10.1371/journal.pone.0146472 (PMC4709000; doi:10.1371/journal.pone.0146472)
Supplement: S2 Table — (DOCX) [file pone.0146472.s002.docx]

Table 2. Vessel Disease Status of Exerciser and Non-exerciser

| **Vessel disease status** | **Exerciser *^a^* (N=330)** | **Non-exerciser (N=366)** | ***P* Value** |
| --- | --- | --- | --- |
| **Coronary stenosis (%)** |  |  | 0.888 |
| 50~74 | 44(13.3) | 44(12.0) |  |
| 75~90 | 133(40.3) | 144(39.3) |  |
| 91~99 | 79(23.9) | 96(26.2) |  |
| 100 | 74(22.4) | 82(22.4) |  |
| **The number of stenosis vessels (N)** |  |  | 0.003 |
| 1 | 121(36.7) | 96(26.2) |  |
| 2 | 79(23.9) | 124(33.9) |  |
| 3 | 130(39.4) | 146(39.9) |  |

Categorical variables expressed as N (%).

^a^ Participants were defined as exercisers if they have done sports-related physical activity, which is a subcategory of physical activity, that is planned, structured, repetitive, and aims to improve or maintain one or more components of physical fitness, for at least 5 years, and still exercised in the recent 3 months before they went to hospital.
