# Supplementary material for: Association between Time of Day of Sports-Related Physical Activity and the Onset of Acute Myocardial Infarction in a Chinese Population
Source: PLoS One. 2016 Jan 11;11(1):e0146472. doi: 10.1371/journal.pone.0146472 (PMC4709000; doi:10.1371/journal.pone.0146472)
Supplement: S3 Table — (DOCX) [file pone.0146472.s003.docx]

Table 3. Characteristics of Time of Day of Sports-related Physical Activity

| **Sports-related**  **physical activity** | **Morning (N=121)** | **Noon**  **(N=6)** | **Afternoon (N=49)** | **Evening (N=158)** | **Early morning (N=32)** | ***P* Value** |
| --- | --- | --- | --- | --- | --- | --- |
| **Intensity *^a^*** |  |  |  |  |  | 0.246 |
| Low | 79(65.3) | 3(50.0) | 37(75.5) | 110(69.6) | 19(59.4) |  |
| Moderate | 20(16.5) | 0(0) | 7(14.3) | 16(10.1) | 6(18.8) |  |
| High | 22(18.2) | 3(50.0) | 5(10.2) | 32(20.3) | 7(21.9) |  |
| **Duration time** |  |  |  |  |  | 0.382 |
| <30 min/day | 10(8.3) | 1(16.7) | 5(10.2) | 11(7.0) | 1(3.1) |  |
| 30~60min/day | 42(34.7) | 3(50.0) | 24(49.0) | 61(38.6) | 9(28.1) |  |
| >60 min/day | 69(57.0) | 2(33.3) | 20(40.8) | 86(54.4) | 22(68.8) |  |
| **Frequency** |  |  |  |  |  | 0.009 |
| <3 times/week | 7(5.8) | 1(16.7) | 4(8.2) | 19(12.0) | 1(3.1) |  |
| 3~5 times/week | 3(2.5) | 1(16.7) | 9(18.4) | 20(12.7) | 2(6.2) |  |
| >5 times/week | 111(91.7) | 4(66.7) | 36(73.5) | 119(75.3) | 29(90.6) |  |

Categorical variables expressed as N (%)

^a^ Intensity of sports-related physical activity was categorized as low (<3METs), moderate (3-6METs) and high (>6METs).
