# Supplementary material for: Association between Time of Day of Sports-Related Physical Activity and the Onset of Acute Myocardial Infarction in a Chinese Population
Source: PLoS One. 2016 Jan 11;11(1):e0146472. doi: 10.1371/journal.pone.0146472 (PMC4709000; doi:10.1371/journal.pone.0146472)
Supplement: S4 Table — (DOCX) [file pone.0146472.s004.docx]

Table 4. Association Between Sports-related Physical Activity and the Onset of AMI.

| **Sports-related**  **physical activity** | **Non-AMI (%)** | **AMI (%)** | **Unadjusted OR (95%CI)** | **Adjusted OR*^c^* (95%CI)** | ***P* Value** |
| --- | --- | --- | --- | --- | --- |
| **Exerciser *^a^*** |  |  |  |  |  |
| No | 146(42.0) | 184(52.9) | 1(reference) | 1(reference) |  |
| Yes | 202(58.0) | 164(47.1) | 0.64(0.48-0.87) | 0.67(0.47-0.94) | 0.023 |
| **Intensity *^b^*** |  |  |  |  |  |
| None | 146(42.0) | 184(52.9) | 1(reference) | 1(reference) |  |
| Low | 131(37.6) | 118(33.9) | 0.71(0.51-0.99) | 0.80(0.55-1.18) | 0.259 |
| Moderate | 26 (7.5) | 22(6.3) | 0.67(0.37-1.23) | 0.60(0.30-1.20) | 0.150 |
| High | 45(12.9) | 24(6.9) | 0.42(0.25-0.73) | 0.39(0.22-0.73) | 0.003 |
| **Duration time** |  |  |  |  |  |
| None | 146(42.0) | 184(52.9) | 1(reference) | 1(reference) |  |
| <30 min/day | 14(4.0) | 14(4.0) | 0.79(0.37-1.72) | 0.81(0.34-1.93) | 0.637 |
| 30~60min/day | 80(23.0) | 59(17.0) | 0.59(0.39-0.87) | 0.65(0.41-1.02) | 0.062 |
| >60 min/day | 108(31.0) | 91(26.1) | 0.67(0.47-0.95) | 0.67(0.45-0.99) | 0.048 |
| **Frequency** |  |  |  |  |  |
| None | 146(42.0) | 184(52.9) | 1(reference) | 1(reference) |  |
| <3 times/week | 11(3.2) | 21(6.0) | 1.52(0.71-3.25) | 1.87(0.79-4.42) | 0.155 |
| 3~5 times/week | 21(6.0) | 14(4.0) | 0.53(0.26-1.08) | 0.94(0.43-2.05) | 0.869 |
| >5 times/week | 170(48.9) | 129(37.1) | 0.60(0.44-0.83) | 0.57(0.39-0.82) | 0.003 |
| **Total EEPA** |  |  |  |  |  |
| None | 146(42.0) | 184(52.9) | 1(reference) | 1(reference) |  |
| 1~450 MET·min/week | 14(4.0) | 22(6.3) | 1.25(0.62-2.52) | 1.64(0.73-3.64) | 0.229 |
| 451~900 MET·min/week | 68(19.5) | 48(13.8) | 0.56(0.36-0.86) | 0.60(0.36-0.97) | 0.039 |
| 901~1350 MET·min/week | 62(17.8) | 67(19.3) | 0.86(0.57-1.29) | 0.88(0.55-1.41) | 0.603 |
| 1351~1800 MET·min/week | 31(8.9) | 18(5.2) | 0.46(0.25-0.86) | 0.45(0.23-0.90) | 0.025 |
| >1800 MET·min/week | 27(7.8) | 9(2.6) | 0.26(0.12-0.58) | 0.18(0.07-0.45) | <0.001 |

Abbreviations: AMI, acute myocardial infarction; OR, odds ratio; CI, confidence interval; EEPA, energy expenditure in physical activity.

^a^ Participants were defined as exercisers if they have done sports-related physical activity, which is a subcategory of physical activity, that is planned, structured, repetitive, and aims to improve or maintain one or more components of physical fitness, for at least 5 years, and still exercised in the recent 3 months before they went to hospital.

^b^ Intensity of sports-related physical activity was categorized as low (<3METs), moderate (3-6METs) and high (>6METs).

^c^ Adjustment for age, sex, smoking status, alcohol use, work-related activity, hypertension, dyslipidemia, diabetes mellitus, family history of CAD, the severity of coronary stenosis and body mass index in the analysis.
