# Supplementary material for: Association between Time of Day of Sports-Related Physical Activity and the Onset of Acute Myocardial Infarction in a Chinese Population
Source: PLoS One. 2016 Jan 11;11(1):e0146472. doi: 10.1371/journal.pone.0146472 (PMC4709000; doi:10.1371/journal.pone.0146472)
Supplement: S5 Table — (DOCX) [file pone.0146472.s005.docx]

Table 5. Association Between Different Times of Day Sports-related Physical Activity and the Onset of AMI.

| **Sports-related**  **physical activity *^a^*** | **Non-AMI (%)** | **AMI (%)** | **Unadjusted OR (95%CI)** | **Adjusted OR *^b^* (95%CI)** | ***P* Value** |
| --- | --- | --- | --- | --- | --- |
| **None** | 146(42.0) | 184(52.9) | 1(reference) | 1(reference) |  |
| **Morning (6:00-10:00)** | 73(21.0) | 48(13.8) | 0.52(0.34-0.80) | 0.60(0.36-0.98) | 0.042 |
| **Noon (10:00~14:00)** | 0(0.0) | 6(1.7) | -- | -- | -- |
| **Afternoon (14:00-18:00)** | 25(7.2) | 24(6.9) | 0.76(0.42-1.39) | 0.87(0.44-1.71) | 0.685 |
| **Evening (18:00-22:00)** | 89(25.6) | 69(19.8) | 0.61(0.42-0.90) | 0.56(0.37-0.87) | 0.009 |
| **Night (22:00-2:00)** | 0(0.0) | 0(0.0) | -- | -- | -- |
| **Early morning (2:00-6:00)** | 15(4.3) | 17(4.9) | 0.90(0.43-1.86) | 0.98(0.43-2.27) | 0.973 |
| **Morning (6:00-10:00)** | 73(21.0) | 48(13.8) | 1(reference) | 1(reference) |  |
| **Evening (18:00-22:00)** | 89(25.6) | 69(19.8) | 1.18(0.73-1.91) | 0.93(0.54-1.64) | 0.824 |

Abbreviations: AMI, acute myocardial infarction; OR, odds ratio; CI, confidence interval.

*^a^* Participants were defined as exercisers if they have done sports-related physical activity, which is a subcategory of physical activity, that is planned, structured, repetitive, and aims to improve or maintain one or more components of physical fitness, for at least 5 years, and still exercised in the recent 3 months before they went to hospital.

*^b^* Adjustment for age, sex, smoking status, alcohol use, work-related activity, hypertension, dyslipidemia, diabetes mellitus, family history of CAD, the severity of coronary stenosis, and body mass index in the analysis.
